# Gastrointestinal Beriberi and Wernicke's Encephalopathy Presented as Chronic Dysphagia and Weight Loss

**DOI:** 10.1002/ccr3.71082

**Published:** 2025-10-03

**Authors:** Bobbi Lee Roth, Yen‐Yi Peng

**Affiliations:** ^1^ Amity Neurology Reno Nevada USA

**Keywords:** diplopia, dysphagia, gastrointestinal beriberi, paresthesia, weight loss, Wernicke's encephalopathy

## Abstract

Chronic dysphagia and weight loss can be presenting signs of gastrointestinal beriberi and Wernicke's encephalopathy.

A 38‐year‐old male presents with a 3‐month history of intractable dysphagia, globus sensation, and an unintentional 80‐pound weight loss (Video 1). A swallowing study revealed oropharyngeal pooling. Initial workup, including neck CT, endoscopy, flexible laryngoscopy, and AChR antibodies, was unremarkable. He reported heavy alcohol use for two decades, consuming approximately two shots of hard liquor and 12 beers three times per week, which predated the onset of dysphagia. Two months after dysphagia began, he experienced generalized numbness. Unrecognized end‐gaze diplopia was noted during the initial interview. His chronic dysphagia significantly improved within days of starting 1000 mg oral thiamine, with full resolution in months. Paresthesia and diplopia also resolved promptly (Video 2) [1].

**VIDEO 1:** On the first day of the consultation, the patient was noted to have end‐gaze diplopia, in addition to 3 months of dysphagia, weight loss, and 1 month of paresthesia affecting the whole body. Video content can be viewed at https://onlinelibrary.wiley.com/doi/10.1002/ccr3.71082.

**VIDEO 2:** On the 8th day of the consultation, after receiving thiamin 500 mg BID, end‐gaze diplopia subsided, and dysphagia and paresthesia improved over several days. On the 44th day, weight loss subsided, and by the 107th day, the patient had recovered well. Video content can be viewed at https://onlinelibrary.wiley.com/doi/10.1002/ccr3.71082.

## Author Contributions

**Bobbi Lee Roth:** validation, writing – review and editing. **Yen‐Yi Peng:** conceptualization, writing – original draft.

## Consent

The authors confirm that written informed consent was obtained from the patient(s) for publication of this case report and accompanying videos/images. A copy of the written consent is available for review by the Editor‐in‐Chief of this journal on request.

## Conflicts of Interest

The authors declare no conflicts of interest.

## Data Availability

The data that support the findings of this study are available on request from the corresponding author. The data are not publicly available due to privacy or ethical restrictions.
